# Supplementary material for: Radiative pumping vs vibrational relaxation of molecular polaritons: a bosonic mapping approach
Source: Nat Commun. 2025 Apr 2;16:3151. doi: 10.1038/s41467-025-58045-5 (PMC11965570; doi:10.1038/s41467-025-58045-5)
Supplement: Supplementary file 1 — Supplementary Information [file 41467_2025_58045_MOESM1_ESM.pdf]

Supplementary Information for “Radiative pumping vs vibrational relaxation of molecular polaritons: a bosonic mapping approach”

Juan B. Pérez-Sánchez and Joel Yuen-Zhou*

Department of Chemistry, University of California San Diego, La Jolla, CA 92093, USA

(Dated: February 13, 2025)

1. BOSONIC MAPPING

Here we explicitly show that imposing permutational symmetries to the many-body vibro-polaritonic wavefunction and focusing on the permutationally-symmetric subspace is equivalent to performing a bosonic mapping where molecules are treated as bosonic particles with internal structure. Although simple derivations of this bosonic mapping are available in the literature [1, 2], here we do so by following a procedure akin to our previous work [3]. First, we derive the equations of motion (EoM) for the coefficients of the Ansatz wavefunction. Second, we apply permutational symmetry conditions. Finally, we notice that the resulting equations of motion are equivalent to those emerging from a bosonic Hamiltonian, where each vibronic state is represented by a bosonic mode and the number of molecules in the vibronic state are given by the excitations in such mode.

The many-body wavefunction for arbitrary number of molecules and excitations (excitons and photons) in the product basis states of uncoupled molecular and photonic eigenstates can be written as

$$|\Psi(t)\rangle = \sum_{J,s,N_{ph}} A_J^{(s,N_{ph})}(t)|\Phi_J^{(s)}, s, N_{ph}\rangle = \sum_{s,N_{ph}} \int d\vec{q}\, \psi^{(s,N_{ph})}(\vec{q},t)|\vec{q}, s, N_{ph}\rangle, \tag{S1}$$

We can write the multidimensional vibrational wavefunction $\psi^{(s,N_{ph})}(\vec{q},t)$ for any given electronic-photonic state as

$$\begin{aligned}\psi^{(s,N_{ph})}(\vec{q},t) &= \sum_J A_J^{(s,N_{ph})}(t)\langle \vec{q}\,|\Phi_J^{(s)}\rangle \\ &= \sum_{J_1 J_2\cdots J_N} A_{J_1 J_2\cdots J_N}^{(s,N_{ph})}(t)\prod_{k=1}^{N}\varphi_{J_k}^{(s_k)}(q_k)\end{aligned} \tag{S2}$$

Assuming the vibrational states $|\varphi_j^{(g/e)}(q)\rangle$ to be eigenstates of $\hat{H}_{mol}$ with eigenvalues $\omega_{g/e,j}$, the ansatz wavefunction of equation (S1) yields the EoM

$$\begin{aligned}i\dot{A}_J^{(s,N_{ph})}(t) &= \omega_J^{(s,N_{ph})}A_J^{(s,N_{ph})}(t) + \sum_{L,s',N'_{ph}}\langle N_{ph}, s; \Phi_J^{(s,N_{ph})}|\hat{H}_I|\Phi_L^{(s',N'_{ph})}; s', N'_{ph}\rangle A_L^{(s',N'_{ph})}(t) \\ &= \omega_J^{(s,N_{ph})}A_J^{(s,N_{ph})}(t) + g\sqrt{N_{ph}+1}\sum_{i=1}^{N}\delta_{s_i,e_i}\sum_{L_i}\langle\varphi_{J_i}^{(e)}|\varphi_{L_i}^{(g)}\rangle A_{J(L_i)}^{(s(g_i),N_{ph}+1)}(t) \\ &+ g\sqrt{N_{ph}}\sum_{i=1}^{N}\delta_{s_i,g_i}\sum_{L_i}\langle\varphi_{J_i}^{(g)}|\varphi_{L_i}^{(e)}\rangle A_{J(L_i)}^{(s(e_i),N_{ph}-1)}(t),\end{aligned} \tag{S3}$$

* joelyuen@ucsd.edu

where the superscript $s(g_i)$ implies that the ith element of the list s has been set to g. The second term in equation (S3) describes absorption while the third one describes emission of a photon by the molecular ensemble.

We exploit permutational symmetries

$$A^{(s_1\cdots s_\kappa\cdots s_\lambda\cdots s_N,N_{ph})}_{J_1\cdots J_\kappa\cdots J_\lambda\cdots J_N}(t) = A^{(s_1\cdots s_\lambda\cdots s_\kappa\cdots s_N,N_{ph})}_{J_1\cdots J_\lambda\cdots J_\kappa\cdots J_N}(t). \tag{S4}$$

We can rewrite all identical amplitudes in terms of those in which the first N_e molecules contain all the electronic excitations and the vibrational states are from lowest to largest vibrational quantum number. Following the same strategy as in one of our previous works [3], can write the EoM starting with a state where all excitations are photons,

$$\begin{aligned} i\dot{A}^{(ee\cdots egg\cdots g,N_{ph})}_{l_1 l_2\cdots l_{N_e}11\cdots 1}(t) &= \left[N_g\omega_{g,1} + N_{ph}\omega_c + \sum_{i=1}^{N_e}\omega'_{e,i}\right] A^{(ee\cdots egg\cdots g,N_{ph})}_{l_1 l_2\cdots l_{N_e}11\cdots 1}(t) \\ &+ g\sqrt{N_{ph}+1}\sum_{i}^{N_e}\sum_{k=1}^{m}\langle\varphi^{(e)}_{l_i}|\varphi^{(g)}_k\rangle A^{(ee\cdots egg\cdots g,N_{ph}+1)}_{l_1\cdots l_{i-1}l_{i+1}\cdots l_{N_e}k11\cdots 1}(t) \\ &+ gN_g\sqrt{N_{ph}}\sum_{l}^{m}\langle\varphi^{(g)}_1|\varphi^{(e)}_l\rangle A^{(ee\cdots egg\cdots g,N_{ph}-1)}_{l_1 l_2\cdots l_{N_e}l11\cdots 1}(t). \end{aligned} \tag{S5}$$

These EoM can be written in terms of coefficients that do not specified the vibrational state of each molecule, but just how many molecules are on each state:

$$A^{(ee\cdots egg\cdots g,N_{ph})}_{l_1 l_2\cdots l_{N_e}j_1 j_2\cdots j_{N_g}}(t) \to A^{(N_{ph})}_{n_1 n_2\cdots n_m,n'_1 n'_2\cdots n'_m}(t), \tag{S6}$$

with $\sum_{i=1}^m n_i = N_g$ and $\sum_{i=1}^m n'_i = N_e$.

These amplitudes can be renormalized by a factor that counts the total number of states that is represented by each coefficient. In general, the coefficient $A^{(N_{ph})}_{n_1 n_2\cdots n_m,n'_1 n'_2\cdots n'_m}(t)$ represents $\frac{N!}{\prod_{i=1}^m n_i! n'_i!}$ different states. Defining renormalized coefficients as

$$\tilde{A}^{(N_{ph})}_{n_1 n_2\cdots n_m,n'_1 n'_2\cdots n'_m}(t) = \sqrt{\frac{N!}{\prod_{i=1}^m n_i! n'_i!}} A^{(N_{ph})}_{n_1 n_2\cdots n_m,n'_1 n'_2\cdots n'_m}(t), \tag{S7}$$

and rewriting the EoM, we get

$$i\dot{\tilde{A}}^{(N_{ph})}_{n_1n_2\cdots n_m,n'_1n'_2\cdots n'_m}(t) = \left[\sum_{i=1}^{m}(n_i\omega_{g,i} + n'_i\omega_{e,i}) + N_{ph}\omega_c\right]\tilde{A}^{(N_{ph})}_{n_1n_2\cdots n_m,n'_1n'_2\cdots n'_m}(t)$$
$$+ g\sqrt{N_{ph}}\sum_{k=1}^{m}\sqrt{n'_k+1}\sum_{k'=1}^{m}\sqrt{n_{k'}}\langle\varphi^{(g)}_{k'}|\varphi^{(e)}_k\rangle\tilde{A}^{(N_{ph}-1)}_{n_1n_2\cdots(n_{k'}-1)\cdots n_m,n'_1n'_2\cdots(n'_k+1)\cdots n'_m}(t)$$
$$+ g\sqrt{N_{ph}+1}\sum_{k=1}^{m}\sqrt{n'_k}\sum_{k'=1}^{m}\sqrt{n_{k'}+1}\langle\varphi^{(e)}_k|\varphi^{(g)}_{k'}\rangle\tilde{A}^{(N_{ph}+1)}_{n_1n_2\cdots(n_{k'}+1)\cdots n_m,n'_1n'_2\cdots(n'_k-1)\cdots n'_m}(t). \quad \text{(S8)}$$

The effective bosonic Hamiltonian that yields the EoM in equation (S8) is given by

$$\hat{H} = \omega_c\hat{a}^\dagger\hat{a} + \sum_i^m\omega_{g,i}\hat{b}_i^\dagger\hat{b}_i + \sum_i^m\omega'_{e,i}\hat{B'}_i^\dagger\hat{B'}_i + g\sum_{ij}^m\langle\varphi^{(e)}_i|\varphi^{(g)}_j\rangle\hat{B'}_i^\dagger\hat{b}_j\hat{a} + h.c., \quad \text{(S9)}$$

where $\hat{a}$, $\hat{b}_i$, and $\hat{B'}_i$ are the bosonic operators that annihilate a photon, a molecule (not an excitation) in the vibronic state $|g,\varphi^{(g)}_i\rangle$, and a molecule in the vibronic state $|e,\varphi^{(e)}_i\rangle$, respectively.

Finally, we move to a frame where the vibrational states $|\varphi^{(g)}_i\rangle$ are used as basis for both ground and excited electronic states via

$$\hat{B}'_i = \sum_j\langle\varphi^{(e)}_i|\varphi^{(g)}_j\rangle\hat{B}_j. \quad \text{(S10)}$$

In this new basis we obtain

$$\hat{H} = \omega_c\hat{a}^\dagger\hat{a} + \sum_i^m\omega_{g,i}\hat{b}_i^\dagger\hat{b}_i + \sum_i^m\omega_{e,i}\hat{B}_i^\dagger\hat{B}_i + \sum_{i\neq j}^m\langle\varphi^{(g)}_i|\hat{V}_{eg}|\varphi^{(g)}_j\rangle\hat{B}_i^\dagger\hat{B}_j + g\sum_i^m\left(\hat{B}_i^\dagger\hat{b}_i\hat{a} + \hat{B}_i\hat{b}_i^\dagger\hat{a}^\dagger\right). \quad \text{(S11)}$$

2. COLLECTIVE STRONG LIGHT-MATTER COUPLING AS BOSONIC STIMULATION TO THE FRANCK-CONDON REGION

Consider a state with all molecules in the *global* ground state and a photon, i.e. $|N00\cdots0,00\cdots0,1\rangle$ (zero temperature). Light-matter interaction at the Franck-Condon (FC) region $g\left(\hat{B}_1^\dagger\hat{b}_1\hat{a} + \hat{B}_1\hat{b}_1^\dagger\hat{a}^\dagger\right)$ is larger ($\propto\sqrt{N}g$) than at any other configuration ($\propto g$), due to bosonic stimulation of the $\hat{b}_1$ mode. When collective strong coupling $g\sqrt{N}$ is reached with a large number of molecules, a perturbative expansion on the light-matter interaction terms away from the FC region gives rise to the $1/N$ expansion of the exact many-body

wavefunction shown in our previous work [3], and to the structure in Fig Supplementary Figure 1.

If more than one molecule can be prepared in the same vibrational state, there can be bosonic stimulation at other nuclear configurations. It is not clear whether thermal states or other ways to prepare vibrationally excited states allow for this phenomenon. On the one hand, the existence of two or more molecules on the same exact vibrational state may be unlikely due to the large dimensionality of the vibrational bath. On the other hand, the vibrationally-excited states might only need to be the same for a finite energy resolution given by the emission timescale. In the main manuscript we assume that significant ($\sim N \gg 1$) bosonic stimulation can only occur at the FC region, and leave the effects of vibrational and exciton-polariton condensation for future works.

3. RADIATIVE PUMPING

The Hamiltonian $\hat{H}_{rp}^{(0)} \equiv \hat{H}_0 + \hat{H}_{vc}$ commutes with the operators $\hat{n}_{i>1} = \hat{b}_{i>1}^{\dagger}\hat{b}_{i>1}$, which represent the number of *ground state* molecules on each vibrational excited state i. This allows us to write the eigenstates of $\hat{H}_{rp}^{(0)}$ in the first excitation manifold as

$$\begin{aligned} |\xi, \{n_j\}\rangle &= a_{\{n_j\}}^{(\xi)} |n_1 n_2 \cdots n_m, 00\cdots 0, 1\rangle + \sum_i^m b_{\{n_j\}}^{(\xi,i)} |(n_1-1)n_2\cdots n_m, \cdots 1_i \cdots, 0\rangle, \\ \hat{H}_{rp}^{(0)}|\xi, \{n_j\}\rangle &= \omega_{\xi,\{n_j\}}|\xi,\{n_j\}\rangle. \end{aligned} \tag{S12}$$

Notice that $n_1 = N - \sum_{i>1} n_i$ can be added as a label for the eigenstates instead of the total number of molecules N.

Although these eigenstates are dressed by *all* vibronic processes (including the slow ones given by W), we can define an initial dark state (which is approximately an eigenstate of $\hat{H}_{rp}^{(0)}$) that corresponds to one excited molecule in a fully Stokes-shifted configuration with negligible overlap with the FC state, e.g.,

$$|ss\rangle = \sum_{i>1}^m c_{exc}^{(i)} |(N-1)0\cdots 0, \cdots 1_i \cdots, 0\rangle = \sum_{i>1}^m c_{exc}^{(i)}|e_i\rangle, \quad \hat{H}_{rp}^{(0)}|ss\rangle = \omega_{ss}|ss\rangle. \tag{S13}$$

The Fermi's Golden Rule rate yields

$$\begin{aligned}\Gamma_{rp} =& 2\pi\sum_{\xi}\sum_{\{n_j\}}|\langle\xi,\{n_j\}|\hat{V}_{rp}|ss\rangle|^2\frac{\gamma_\xi/\pi}{(\omega_{\xi,\{n_j\}}-\omega_{ss})^2+\gamma_\xi^2}\\ =& 2\pi g^2\sum_{\xi}\sum_{j>1}^{m}|a_{1_j}^{(\xi)}|^2|c_{exc}^{(j)}|^2\frac{\gamma_\xi/\pi}{(\omega_\xi-(\omega_{ss}-\omega_{g,j}))^2+\gamma_\xi^2},\end{aligned} \tag{S14}$$

recovering Equation 9 in the main text. Here we renamed $a^{(\xi)}_{(N-1)\cdots 1_j\cdots}\equiv a^{(\xi)}_{1_j}$, set $\omega_{g,1}=0$, and approximated $\omega_{\xi,(N-1)\cdots 1_j\cdots}\approx\omega_\xi+\omega_{g,j}$, with $\omega_\xi=\omega_{\xi,(N-1)\cdots 0\cdots}$ being the polariton frequency and $\omega_{g,j}$ being the frequency of the phonon created during the emission. This approximation is valid in the large N limit, as shown in the last section of this SI. The approximation implies that the polariton Rabi splitting does not significantly change when one of the ground state molecules has a phonon ($g\sqrt{N}\approx g\sqrt{N-1}$), and the polariton energies are only shifted by such phonon frequency. We also renamed $a^{(\xi)}_{(N-1)\cdots 1_j\cdots}=a^{(\xi)}_{1_j}$, and used similar simplifications in the notation throughout the manuscript.

3.1. Radiative pumping from linear optics

Here, we show that the radiative pumping formula in Equation 11 of the main text, written in terms of linear optical properties, is equivalent to equation (S14) above,

$$\begin{aligned}\Gamma_{rp} &= \int d\omega\Gamma_{rp}(\omega)=\frac{2g^2}{\kappa}\int d\omega\sigma_{em}(\omega)\left[A(\omega)+2T(\omega)\right]\\ &= -2g^2\int d\omega\sigma_{em}(\omega)\mathrm{Im}\left[D^R(\omega)\right]\\ &= 2\pi g^2\sum_{\xi}|a_{1_j}^{(\xi)}|^2\int d\omega\sigma_{em}(\omega)\frac{\gamma_\xi/\pi}{(\omega-\omega_\xi)^2+\gamma_\xi^2}\\ &= 2\pi g^2\sum_{\xi}\sum_{j}^{m}|a_{1_j}^{(\xi)}|^2|c_{exc}^{(j)}|^2\int d\omega\delta(\omega-(\omega_{ss}-\omega_{g,j}))\frac{\gamma_\xi/\pi}{(\omega-\omega_\xi)^2+\gamma_\xi^2}\\ &= 2\pi g^2\sum_{\xi}\sum_{j}^{m}|a_{1_j}^{(\xi)}|^2|c_{exc}^{(j)}|^2\frac{\gamma_\xi/\pi}{(\omega_\xi-(\omega_{ss}-\omega_{g,j})))^2+\gamma_\xi^2},\end{aligned} \tag{S15}$$

where we used the following results:

First, we write the photon density of states in terms of the photon-photon correlation

function $D^{(R)}(\omega)$ in the $N \to \infty$ limit,

$$\begin{aligned} D^{(R)}(\omega) &= \langle N00\cdots 0, 00\cdots 0, 1| \frac{1}{\omega - \hat{H}_{rp}^{(0)}} |N00\cdots 0, 00\cdots 0, 1\rangle \\ &= \sum_{\xi} |a_0^{(\xi)}|^2 \frac{1}{\omega - \omega_\xi + i\gamma_\xi} \\ &\approx \sum_{\xi} |a_{1_j}^{(\xi)}|^2 \frac{1}{\omega - \omega_\xi + i\gamma_\xi} \\ \mathrm{Im}\left[D^{(R)}(\omega)\right] &\approx -\sum_{\xi} |a_{1_j}^{(\xi)}|^2 \frac{\gamma_\xi}{(\omega - \omega_\xi)^2 + \gamma_\xi^2}. \end{aligned} \tag{S16}$$

Here we added a broadening γ_ξ to the eigenvalues of $\hat{H}_{rp}^{(0)}$ due to finite cavity lifetime κ. We also simplified the notation for the photonic Hopfield coefficient as $a_{N\cdots 0\cdots}^{(\xi)} = a_0^{(\xi)}$ and $a_{(N-1)\cdots 1_j\cdots}^{(\xi)} = a_{1_j}^{(\xi)}$, and set $\omega_{g,1} = 0$. The third line comes about from approximating $g\sqrt{N-1} \approx g\sqrt{N}$ (see Supplementary Section 9 for details). This approximation is valid for large number of molecules where the contraction of the Rabi splitting is negligible compared to the broadening of the spectra.

Second, we express $\mathrm{Im}\left[D^{(R)}(\omega)\right]$ in terms of the polariton linear response equations in Refs. [4–6]

$$-\mathrm{Im}\left[D^{R}(\omega)\right] = \frac{1}{2}\left[\left(\frac{\kappa}{\kappa_R \kappa_L}\right) T(\omega) + \frac{1}{\kappa_L} A(\omega)\right] = \frac{2T(\omega) + A(\omega)}{\kappa}, \tag{S17}$$

where we assumed $\kappa_L = \kappa_R = \kappa/2$, and $A(\omega)$ and $T(\omega)$ are the polariton absorption and transmission spectra, in the $N \to \infty$ limit, respectively.

4. DARK STATES AS INCOHERENT EXCITONS VS DARK STATES OF THE TC MODEL

Let us consider the states with one molecule in the excited modes $\hat{B}_1$ or $\hat{B}_{i>1}$, while the rest are in the global ground state, i.e., $|e_1\rangle = |(N-1)0\cdots 0, 10\cdots 0, 0\rangle$ and $|e_{i>1}\rangle = |(N-1)0\cdots 0, 0\cdots 1_i \cdots 0, 0\rangle$. The vibronic many-body wavefunction represented by $|e_1\rangle$ can be written in first quantization as a product state of a totally symmetric excitonic state, a vibrational state, and a photon state, while the vibronic many-body wavefunction represented by $|e_{i>1}\rangle$ is an entangled state of the electronic and vibrational degrees of freedom,

$$|\Psi_{e_1}\rangle = \frac{1}{\sqrt{N}}\left(\sum_j^N |gg\cdots e^{(j)}\cdots g\rangle\right)\left(\prod_k^N |\varphi_1^{(g)}\rangle\right)|0\rangle = \left(\frac{1}{\sqrt{N}}\sum_j^N |e_j\rangle\right)\left(\prod_{j'}^N |\varphi_1^{(g)}\rangle\right)|0\rangle$$

$$|\Psi_{e_i}\rangle = \frac{1}{\sqrt{N}}\left(\sum_j^N |e^{(j)}\varphi_i^{(g)}\rangle \otimes_{j'\neq j}^N |g\varphi_1^{(g)}\rangle\right)|0\rangle. \tag{S18}$$

By tracing out the photonic and vibrational degrees of freedom we obtain the electronic reduced density matrices for the states $|e_i\rangle$,

$$\rho_{elec,1} = \mathrm{Tr}_{vib,ph}\left[|\Psi_{e_1}\rangle\langle\Psi_{e_1}|\right] = \begin{pmatrix} 1 & 0 & \cdots & 0 \\ 0 & 0 & \cdots & 0 \\ \vdots & \vdots & \ddots & \vdots \\ 0 & 0 & \cdots & 0 \end{pmatrix} \quad \rho_{elec,i>1} = \mathrm{Tr}_{vib,ph}\left[|\Psi_{e_i}\rangle\langle\Psi_{e_i}|\right] = \frac{1}{N}\begin{pmatrix} 1 & 0 & \cdots & 0 \\ 0 & 1 & \cdots & 0 \\ \vdots & \vdots & \ddots & \vdots \\ 0 & 0 & \cdots & 1 \end{pmatrix}, \tag{S19}$$

where we have used the Fourier basis for the electronic states

$$|B\rangle = \frac{1}{\sqrt{N}}\sum_j |e_j\rangle,$$
$$|D_k\rangle = \frac{1}{\sqrt{N}}\sum_j e^{-i2\pi kj/N}|e_j\rangle, \quad k = 1, \ldots, N-1. \tag{S20}$$

The dark states in the radiative pumping regime are not equivalent to the dark states $|D_k\rangle$ of the Tavis-Cummings Hamiltonian, although they keep certain resemblance: an excitation in the FC configuration represents a pure bright state of the TC Hamiltonian, while an excitation far from the FC region is a mixture of all the dark states with only $1/N$ contribution from the bright state. Since Stokes-shifted molecules can still couple to the cavity mode via single-molecule light-matter coupling (which is the perturbation in this regime), these dark states can relax into polaritons via fluorescence.

5. VIBRATIONAL RELAXATION

The Hamiltonian $\hat{H}_{vr}^{(0)} \equiv \hat{H}_0 + \hat{H}_{sm}$ in the weak vibronic coupling limit can be written as

$$\hat{H}_{vr}^{(0)} = \omega_c \hat{a}^\dagger \hat{a} + \sum_i^m \omega_{g,i}\hat{b}_i^\dagger \hat{b}_i + \sum_i^m \omega_{e,i}\hat{B}_i^\dagger \hat{B}_i + g\sum_i^m \left(\hat{B}_i^\dagger \hat{b}_i \hat{a} + \hat{B}_i \hat{b}_i^\dagger \hat{a}^\dagger\right). \tag{S21}$$

All vibronic couplings are absent from $\hat{H}_{vr}^{(0)}$ since they are considered perturbations. For simplicity we restrict ourselves to the case where $\langle\varphi_i^{(g)}|\hat{V}_e|\varphi_i^{(g)}\rangle = \langle\varphi_i^{(g)}|\hat{V}_g|\varphi_i^{(g)}\rangle + \omega_0$, e.g., the linear-vibronic coupling model [7].

The Hamiltonian $\hat{H}_{vr}^{(0)}$ commutes with the operators $\hat{n}_i + \hat{n'}_i = \hat{b}_i^\dagger \hat{b}_i + \hat{B}_i^\dagger \hat{B}_i$, which represent the number of molecules on each vibrational excited state i (regardless of their electronic state). We focus on the first excitation manifold, where $\sum_i n'_i = 1$, $n'_i \in \{0,1\}$. We can write the Hamiltonian $\hat{H}_{rp}^{(0)}$ projected on particular set of quantum numbers $\{n_i + n'_i\}$ as

$$\mathbf{H}^{(0)}_{\mathbf{vc},\{\mathbf{n_i}+\mathbf{n'_i}\}} = \begin{pmatrix} \sum_i^m n_i\omega_{g,i} + \omega_c & g\sqrt{n_1} & g\sqrt{n_2} & \cdots & g\sqrt{n_m} \\ g\sqrt{n_1} & \sum_i^m n_i\omega_{g,i} + \omega_0 & 0 & \cdots & 0 \\ g\sqrt{n_2} & 0 & \sum_i^m n_i\omega_{g,i} + \omega_0 & \cdots & 0 \\ \vdots & \vdots & \vdots & \ddots & \vdots \\ g\sqrt{n_m} & 0 & 0 & \cdots & \sum_i^m n_i\omega_{g,i} + \omega_0 \end{pmatrix}, \tag{S22}$$

The eigenstates of $\hat{H}_{vc}^{(0)}$ can be separated exactly into polaritons and dark states. The polariton states can be written as

$$|\xi_\pm, \{n_j\}\rangle = c^{(ph)}_{\xi_\pm,\{n_j\}}|n_1 n_2 \cdots n_m, 00\cdots 0, 1\rangle + \sum_i^m c^{(exc,i)}_{\xi_\pm,\{n_j\}}|\cdots(n_i-1)\cdots,\cdots 1_i \cdots, 0\rangle$$
$$\omega_{\xi_\pm,\{n_j\}} = \sum_i^m n_i\omega_{g,i} + \omega_0 + \frac{1}{2}\left(\Delta \pm 2g\sqrt{N}\right), \tag{S23}$$

with

$$c^{(ph)}_{\xi_\pm,\{n_j\}} = \frac{\Delta \pm \sqrt{4g^2N + \Delta^2}}{\sqrt{4g^2N + (\Delta \pm \sqrt{4g^2N+\Delta^2})^2}}$$
$$c^{(exc,i)}_{\xi_\pm,\{n_j\}} = \frac{2g\sqrt{n_i}}{\sqrt{4g^2N + (\Delta \pm \sqrt{4g^2N+\Delta^2})^2}}. \tag{S24}$$

To calculate the Fermi's Golden Rule (FGR) rate at zero temperature we start from a dark initial state, which must have at least a single phonon in the vibrational state k. i.e., $n_1 + n'_1 = N-1$, $n_k + n'_k = 1$. This dark state is obtained from diagonalizing the Hamiltonian

$$\mathbf{H}^{(0)}_{\mathbf{vc},(\mathbf{N-1})\cdots\mathbf{1_k}\cdots} = \begin{pmatrix} \sum_i^m n_i\omega_{g,i} + \omega_c & g\sqrt{N-1} & g \\ g\sqrt{N-1} & \sum_i^m n_i\omega_{g,i} + \omega_0 & 0 \\ g & 0 & \sum_i^m n_i\omega_{g,i} + \omega_0 \end{pmatrix}, \tag{S25}$$

which in the particle notation yields

$$|D_k\rangle = \sqrt{\frac{N-1}{N}}|e_k\rangle - \frac{1}{\sqrt{N}}|g_k e_1\rangle. \tag{S26}$$

Notice that k corresponds to a vibrational *state* of a multidimensional PES, and not to a vibrational *mode*. This dark state is an admixture of one-particle (first term) and two-particle (second term) states considered by Herrera and Spano [8]. From this dark state, the vibrational relaxation rate into polariton states (eigenstates of $\hat{H}^{(0)}$ with non-zero photonic component) yields

$$\begin{aligned}
\Gamma_{\xi_\pm \leftarrow D_k} &= 2\pi \sum_{\{n_j\}} |\langle \xi_\pm, \{n_j\}|\hat{V}_{vr}|D_k\rangle|^2 \frac{\gamma_{\xi_\pm}/\pi}{(\omega_{\xi_\pm,\{n_j\}} - ((N-1)\omega_{g,1} + \omega_{e,k}))^2 + \gamma_{\xi_\pm}^2} \\
&= 2\pi \left(\frac{N-1}{N}\right) |c^{(exc,1)}_{\xi_\pm, N\cdots 0\cdots}|^2 |V_{eg,1k}|^2 \frac{\gamma_{\xi_\pm}/\pi}{(\omega_{g,1} - \omega_{g,k} + \frac{1}{2}(\Delta \pm 2g\sqrt{N}))^2 + \gamma_{\xi_\pm}^2} \\
&+ 2\pi \left(\frac{N-1}{N}\right) \sum_{i>1\neq k}^{m} |c^{(exc,i)}_{\xi_\pm,(N-1)\cdots 1_i \cdots}|^2 |V_{eg,ik}|^2 \frac{\gamma_{\xi_\pm}/\pi}{(\omega_{g,i} - \omega_{g,k} + \frac{1}{2}(\Delta \pm 2g\sqrt{N}))^2 + \gamma_{\xi_\pm}^2} \\
&+ 2\pi \left(\frac{1}{N}\right) \sum_{i>1\neq k}^{m} |c^{(exc,i)}_{\xi_\pm,(N-2)\cdots 1_i \cdots 1_k \cdots}|^2 |V_{eg,i1}|^2 \frac{\gamma_{\xi_\pm}/\pi}{(\omega_{g,i} - \omega_{g,1} + \frac{1}{2}(\Delta \pm 2g\sqrt{N}))^2 + \gamma_{\xi_\pm}^2} \\
&+ 2\pi \left(\frac{1}{N}\right) |c^{(exc,k)}_{\xi_\pm,(N-2)\cdots 2_k \cdots}|^2 |V_{eg,k1}|^2 \frac{\gamma_{\xi_\pm}/\pi}{(\omega_{g,k} - \omega_{g,1} + \frac{1}{2}(\Delta \pm 2g\sqrt{N}))^2 + \gamma_{\xi_\pm}^2}.
\end{aligned} \tag{S27}$$

To interpret the results more easily, let's assume $\Delta = 0$, which yields

$$|c^{(exc,i)}_{\xi_\pm,(N-2)\cdots 1_i \cdots 1_k \cdots}|^2 = \frac{1}{2N}. \tag{S28}$$

The dark-to-polariton rate yields

$$\begin{aligned}
\Gamma_{\xi_\pm \leftarrow D_k} &= 2\pi \left(\frac{N-1}{N}\right)\left(\frac{1}{2}\right) |V_{eg,1k}|^2 \frac{\gamma_{\xi_\pm}/\pi}{(\omega_{g,1} - \omega_{g,k} \pm g\sqrt{N})^2 + \gamma_{\xi_\pm}^2} \\
&+ 2\pi \left(\frac{N-1}{N}\right)\left(\frac{1}{2N}\right) \sum_{i>1\neq k}^{m} |V_{eg,ik}|^2 \frac{\gamma_{\xi_\pm}/\pi}{(\omega_{g,i} - \omega_{g,k} \pm g\sqrt{N})^2 + \gamma_{\xi_\pm}^2} \\
&+ 2\pi \left(\frac{1}{N}\right)\left(\frac{1}{2N}\right) \sum_{i>1\neq k}^{m} |V_{eg,i1}|^2 \frac{\gamma_{\xi_\pm}/\pi}{(\omega_{g,i} - \omega_{g,1} \pm g\sqrt{N})^2 + \gamma_{\xi_\pm}^2} \\
&+ 2\pi \left(\frac{1}{N}\right)\left(\frac{1}{N}\right) |V_{eg,k1}|^2 \frac{\gamma_{\xi_\pm}/\pi}{(\omega_{g,k} - \omega_{g,1} \pm g\sqrt{N})^2 + \gamma_{\xi_\pm}^2}.
\end{aligned} \tag{S29}$$

Here, the first term corresponds to couplings from Stokes-shifted configurations directly into the FC region (recurrences), and the fourth term corresponds to the release of a phonon in

the vibrational state k (same as the first molecule). This latter process in unlikely if many vibrational modes per molecule are present. Ignoring these two terms we obtain

$$\Gamma_{\xi_\pm \leftarrow D_k} = 2\pi\left(\frac{N-1}{N}\right)\left(\frac{1}{2N}\right)\sum_{i>1\neq k}^{m}|V_{eg,ik}|^2\frac{\gamma_{\xi_\pm}/\pi}{(\omega_{g,i}-\omega_{g,k}\pm g\sqrt{N})^2+\gamma_{\xi_\pm}^2}$$
$$+2\pi\left(\frac{1}{N}\right)\left(\frac{1}{2N}\right)\sum_{i>1\neq k}^{m}|V_{eg,i1}|^2\frac{\gamma_{\xi_\pm}/\pi}{(\omega_{g,i}-\omega_{g,1}\pm g\sqrt{N})^2+\gamma_{\xi_\pm}^2}. \tag{S30}$$

6. UNDERSTANDING VIBRATIONAL RELAXATION IN THE WEAK VIBRONIC COUPLING REGIME

To understand which processes are involved in the vibrational relaxation rate in the weak vibronic coupling regime, we can analyze the initial dark state and final polariton states involved in the rate. The initial dark state is given in equation (S26). The final polariton state that gives rise to the term proportional to $\frac{N-1}{2N^2}$ is

$$|\xi_\pm,(N-1)\cdots 1_i\cdots\rangle = \frac{1}{\sqrt{2}}|g_i 1\rangle + \frac{1}{\sqrt{2}}\sqrt{\frac{N-1}{N}}|g_i e_1\rangle + \frac{1}{\sqrt{2}}\sqrt{\frac{1}{N}}|e_i\rangle, \tag{S31}$$

and its contribution to the rate can be understood due to the pathway $|e_k\rangle \xrightarrow{W} |e_i\rangle \xrightarrow{g} |g_i,1\rangle$, which is simply Stokes shift followed by emission. Notice that this process creates phonons in a single ground state molecule; the one that emits.

The final polariton state that gives rise to the term proportional to $\frac{1}{2N^2}$ is

$$|\xi_\pm,(N-2)\cdots 1_i\cdots 1_k\cdots\rangle = \frac{1}{\sqrt{2}}|g_i g_k 1\rangle + \frac{1}{\sqrt{2}}\sqrt{\frac{N-2}{N}}|g_i g_k e_1\rangle + \frac{1}{\sqrt{2}}\sqrt{\frac{1}{N}}|g_k e_i\rangle$$
$$+\frac{1}{\sqrt{2}}\sqrt{\frac{1}{N}}|g_i e_k\rangle, \tag{S32}$$

and its contribution to the rate can be understood due to the pathway $|e_k\rangle \xrightarrow{g} |g_k 1\rangle \xrightarrow{g\sqrt{N-1}} |g_k e_1\rangle \xrightarrow{W} |g_k e_i\rangle \xrightarrow{g} |g_k g_i 1\rangle$, which requires collective strong coupling as well as two actions of the single-molecule light-matter coupling term g. This process creates phonons in two ground state molecules; one via emission and another via Raman scattering. This is why we call it polariton-assisted Raman scattering. It resembles the mechanism called vibrationally-assisted scattering in multimode cavities. We describe its rate and mechanism more formally in the next section.

7. UNDERSTANDING VIBRATIONAL RELAXATION IN THE STRONG VIBRONIC COUPLING REGIME

A more transparent way to understand high-order processes in the single-molecule light-matter coupling g arises when partitioning the total Hamiltonian as $\hat{H} = \hat{H}_{rp}^{(0)} + \hat{H}_{sm}$. $\hat{H}_{rp}^{(0)}$ commutes with the operators $\hat{n}_{i>1} = \hat{b}_{i>1}^{\dagger}\hat{b}_{i>1}$ (the number of ground state molecules in the vibrationally excited state i), while the single-molecule light-matter coupling Hamiltonian $\hat{H}_{sm}$ breaks such symmetry. This gives rise to the structure shown in Fig. Supplementary Figure 1, which is at the core of CUT-E [3]. Interestingly, since $\hat{H}_{rp}^{(0)}$ commutes with the number of excitations $\hat{N}_{exc} = \hat{a}^{\dagger}\hat{a} + \sum_i^m \hat{B}_i^{\dagger}\hat{B}_i$, we can define the "number of particles" $\hat{N}_p = \hat{a}^{\dagger}\hat{a} + \sum_i^m \hat{B}_i^{\dagger}\hat{B}_i + \sum_{i>1}^m \hat{n}_i$ as another good quantum number. It counts the sum of the number of photons, molecules electronically excited, and molecules vibrationally excited. This connects our bosonic formalism with previous works by Herrera and Spano [8, 9].

By looking at the dark and polariton states in the weak vibronic coupling regime (equation (S23)), we can easily check that they are delocalized over only two CUT-E boxes. This means that vibrational relaxation can occur via two actions of g at most (first order in the initial state and first order in the final state). However, when large vibronic coupling regime is included in $\hat{H}_0$, $\hat{n}_{i>1}$ no longer commutes with $\hat{H}_{rp}^{(0)}$, the eigenstates are delocalized over the entire CUT-E hierarchy, and all possible actions of g become relevant for the vibrational relaxation rate. Yet, we can define radiative pumping and polariton-assisted Raman scattering using the diagram in Fig. Supplementary Figure 1. Starting with an incoherent exciton $|ss\rangle$ and zero ground state molecules with phonons (first box), radiative pumping is the rate of going from the first to the second box (creating phonons in the molecule that emits). On the other hand, the polariton-assisted Raman scattering is the rate of going from the first box to the third box, creating phonons in two molecules. States in the second box act as intermediate states for resonant and non-resonant scattering processes.

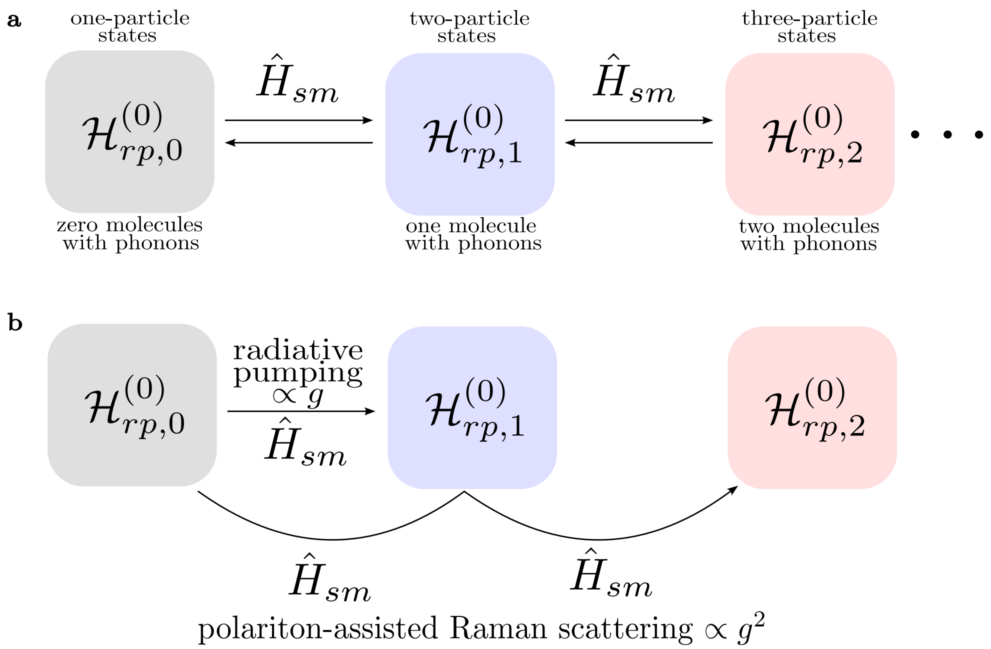

Supplementary Figure 1. Representation of radiative pumping and polariton-assisted Raman scattering using CUT-E. a) Structure of Hamiltonian arising by partitioning the molecular polariton Hamiltonian as $\hat{H} = \hat{H}^{(0)}_{rp} + \hat{H}_{sm}$, with $\hat{H}_{sm}$ containing all single-molecule light-matter coupling terms ($\langle \hat{H}_{sm} \rangle \sim g$). The CUT-E "box" for $\mathcal{H}^{(0)}_{rp,M}$ represents the subspace of $\mathcal{H}^{(0)}_{rp}$ with $M = \sum_{i>1}^{m} n_i$, where M is the the number of ground state molecules with phonons. The first box contains all one-particle states (one exciton/photon), the second box contains all two-particle states (one exciton/photon + one molecule with vibrational excitations), the third box contains all three-particle states (one exciton/photon + two molecules with vibrational excitations), and so on. b) Radiative pumping and polariton-assisted Raman scattering.

8. CALCULATING RADIATIVE PUMPING AND POLARITON-ASSISTED RAMAN SCATTERING RATES

The polariton-assisted Raman scattering rate from second-order perturbation theory yields

$$\Gamma_{scatt} = 2\pi \sum_{\xi',\{n'_j\}} |A_{\xi',\{n'_j\}\leftarrow ss}|^2 \frac{\gamma_{\xi'}/\pi}{(\omega_{\xi',\{n'_j\}} - \omega_{ss})^2 + \gamma^2_{\xi'}},$$

$$A_{\xi',\{n'_j\}\leftarrow ss} = \sum_{\xi,\{n_j\}} \frac{\langle \xi', \{n'_j\}|\hat{H}_{sm}|\xi, \{n_j\}\rangle\langle \xi, \{n_j\}|\hat{H}_{sm}|ss\rangle}{\omega_{\xi,\{n_j\}} - \omega_{ss} + i\gamma_\xi}, \tag{S33}$$

where $|\xi, \{n_j\}\rangle$ are the eigenstates of $\hat{H}_{rp}^{(0)}$. Plugging equations (S12) and (S13) into equation (S33), and summing over all final states with two ground-state molecules with phonons, we obtain

$$\Gamma_{scatt} = 2\pi \sum_{\xi', i \geq j > 1} |A_{\xi', 1_i, 1_j \leftarrow ss}|^2 \frac{\gamma_{\xi'}/\pi}{(\omega_{\xi'} + \omega_{\nu_j} - (\omega_{ss} - \omega_{\nu_i}))^2 + \gamma_{\xi'}^2}, \tag{S34}$$

where we sum over all final states with phonons in two ground-state molecules, and

$$\begin{aligned} A_{\xi', 1_i, 1_j \leftarrow ss} =& g^2 \sum_{\xi} \left(\frac{a_{1_i 1_j}^{(\xi')*} b_{1_i}^{(\xi, j)} a_{1_i}^{(\xi)*} c_{exc}^{(i)}}{\omega_\xi - (\omega_{ss} - \omega_{\nu_i}) + i\gamma_\xi} + \frac{a_{1_i 1_j}^{(\xi')*} b_{1_j}^{(\xi, i)} a_{1_j}^{(\xi)*} c_{exc}^{(j)}}{\omega_\xi - (\omega_{ss} - \omega_{\nu_j}) + i\gamma_\xi} \right) \text{ for } i \neq j \\ A_{\xi', 1_i 1_i \leftarrow ss} =& A_{\xi', 2_i \leftarrow ss} = \sqrt{2} g^2 \sum_{\xi} \left(\frac{a_{2_i}^{(\xi')*} b_{1_i}^{(\xi, i)} a_{1_i}^{(\xi)*} c_{exc}^{(i)}}{\omega_\xi - (\omega_{ss} - \omega_{\nu_i}) + i\gamma_\xi} \right) \text{ for } i = j. \end{aligned} \tag{S35}$$

Exploiting the symmetries of $\hat{H}_{rp}^{(0)}$ mentioned in the previous section, the Hamiltonian $\hat{H}_{rp}^{(0)}$ can be written as

$$\hat{H}_{rp}^{(0)} = \bigoplus_{M=0}^{N} \hat{H}_{rp,M}^{(0)}. \tag{S36}$$

Notice that, since the excitation in the ground electronic state can be in any vibrational state m, we can write

$$\hat{H}_{rp,1}^{(0)} = \bigoplus_{i>1}^{m} \hat{H}_{rp,1_i}^{(0)}. \tag{S37}$$

Now we can explicitly write the matrices for the first and second blocks of $\hat{H}_{rp}^{(0)}$ in the many-body vibronic basis as.

$$\mathbf{H_{rp,0}^{(0)}} = \begin{pmatrix} \omega_c & g\sqrt{N} & 0 & 0 & \cdots & 0 \\ g\sqrt{N} & \omega_{e,1} & V_{eg,12} & 0 & \cdots & 0 \\ 0 & V_{eg,21} & \omega_{e,2} & V_{eg,23} & \cdots & 0 \\ 0 & 0 & V_{eg,32} & \omega_{e,3} & \cdots & 0 \\ \vdots & \vdots & \vdots & \vdots & \ddots & \vdots \\ 0 & 0 & 0 & 0 & \cdots & \omega_{e,m} \end{pmatrix}, \tag{S38}$$

$$\mathbf{H}^{(\mathbf{0})}_{\mathbf{rp},\mathbf{1_{i>1}}} = \begin{pmatrix} \omega_c+\omega_{g,i} & g\sqrt{N-1} & 0 & & \cdots & 0 \\ g\sqrt{N-1} & \omega_{e,1}+\omega_{g,i} & V_{eg,12} & 0 & \cdots & 0 \\ 0 & V_{eg,21} & \omega_{e,2}+\omega_{g,i} & V_{eg,23} & \cdots & 0 \\ 0 & 0 & V_{eg,32} & \omega_{e,3}+\omega_{g,i} & \cdots & 0 \\ \vdots & \vdots & \vdots & \vdots & \ddots & \vdots \\ 0 & 0 & 0 & 0 & \cdots & \omega_{e,m}+\omega_{g,i} \end{pmatrix}. \tag{S39}$$

It is clear that, in the $N \gg 1$ limit, the eigenstates of $\hat{H}^{(0)}_{rp,0}$ and $\hat{H}^{(0)}_{rp,1}$ are identical, although the eigenvalues of the latter are shifted by a vibrational frequency $\omega_{g,i}$. This allows us to make the approximations $\omega_{\xi,(N-1)\cdots 1_i\cdots} = \omega_\xi + \omega_{g,i}$, $a^{(\xi)}_{(N-2)\cdots 1_i \cdots 1_j \cdots} = a^{(\xi)}_{(N-1)\cdots 1_i\cdots} = a^{(\xi)}_{N\cdots 0\cdots}$, and $b^{(\xi,j)}_{(N-1)\cdots 1_i\cdots} = b^{(\xi,j)}_{N\cdots 0\cdots}$. In other words, only $\hat{H}^{(0)}_{rp,0}$ must be diagonalized to obtain Γ_{scatt}.

9. POTENTIAL ENERGY SURFACES FOR NUMERICAL SIMULATIONS

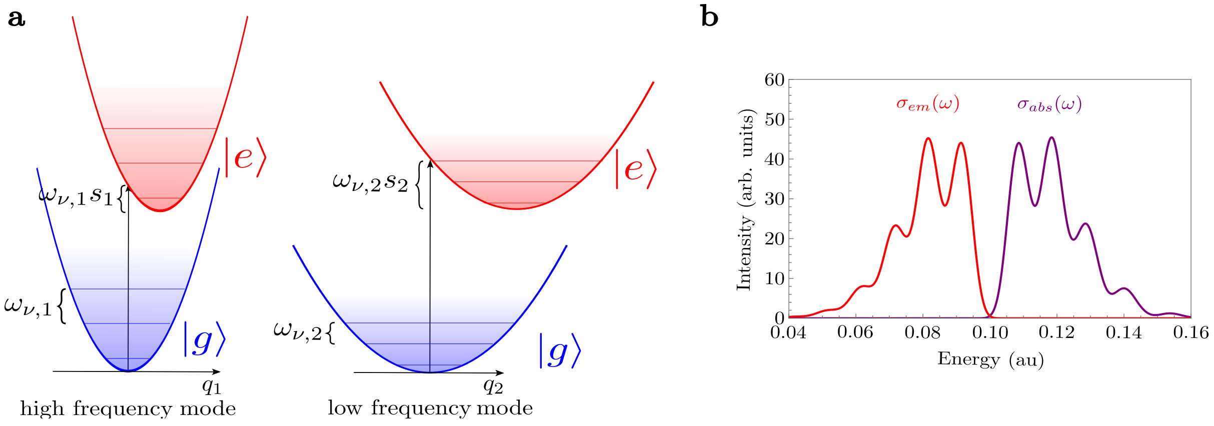

Supplementary Figure 2. Potential energy surfaces used in the numerical calculations. a) our molecular Hamiltonian considers two electronic states and two vibrational modes with strong vibronic couplings. The Stokes-shifted state $|ss\rangle$ corresponds to the lowest energy eigenstate of in the excited electronic state. b) Emission and absorption spectra of the bare molecules.

SUPPLEMENTARY REFERENCES

[1] Silva, R. E. F. & Feist, J. Permutational symmetry for identical multilevel systems: A second-quantized approach. *Phys. Rev. A* **105**, 043704 (2022).

[2] Pérez-Sánchez, J. B., Koner, A., Raghavan-Chitra, S. & Yuen-Zhou., J. CUT-E as a $1/N$ expansion for multiscale molecular polariton dynamics. Preprint at https://arxiv.org/abs/2410.14175 (2024).

[3] Pérez-Sánchez, J. B., Koner, A., Stern, N. P. & Yuen-Zhou, J. Simulating molecular polaritons in the collective regime using few-molecule models. *Proc. Natl. Acad. Sci. USA.* **120**, e2219223120 (2023).

[4] Ćwik, J. A., Kirton, P., De Liberato, S. & Keeling, J. Excitonic spectral features in strongly coupled organic polaritons. *Phys. Rev. A* **93**, 033840 (2016).

[5] Zeb, M. A., Kirton, P. G. & Keeling, J. Exact states and spectra of vibrationally dressed polaritons. *ACS Photonics* **5**, 249–257 (2018).

[6] Yuen-Zhou, J. & Koner, A. Linear response of molecular polaritons. *J. Chem. Phys.* **160**, 154107 (2024).

[7] Litinskaya, M., Reineker, P. & Agranovich, V. Fast polariton relaxation in strongly coupled organic microcavities. *J. Lumin.* **110**, 364–372 (2004).

[8] Herrera, F. & Spano, F. C. Dark vibronic polaritons and the spectroscopy of organic microcavities. *Phys. Rev. Lett.* **118**, 223601 (2017).

[9] Spano, F. C. Exciton–phonon polaritons in organic microcavities: Testing a simple ansatz for treating a large number of chromophores. *J. Chem. Phys.* **152**, 204113 (2020).
